# Hepatic polypeptide nutrient solution improves high‐cholesterol diet‐induced rats with nonalcoholic fatty liver disease by activating AMP‐activated protein kinase signaling pathway

**DOI:** 10.1002/fsn3.3990

**Published:** 2024-02-01

**Authors:** Yingying Xiao, Jianan Wang, Ying Zhang, Ting Zhang, Xingzhong Qi, Lei Hou, Zhihong Ma, Feng Xu

**Affiliations:** ^1^ School of Basic Medicine Hebei University of Chinese Medicine Shijiazhuang Hebei China; ^2^ Graduate School Hebei University of Chinese Medicine Shijiazhuang Hebei China; ^3^ Experimental Center Hebei University of Chinese Medicine Shijiazhuang Hebei China; ^4^ Hebei Zhitong Biological Pharmaceutical Co., Ltd. Baoding Hebei China; ^5^ Hebei International Cooperation Center for Ion Channel Function and Innovative Traditional Chinese Medicine Shijiazhuang Hebei China; ^6^ Hebei Key Laboratory of Integrative Medicine on Liver‐Kidney Patterns Shijiazhuang Hebei China

**Keywords:** AMPK, hepatic polypeptide nutrient solution, lipid metabolism, NAFLD

## Abstract

Hepatic polypeptide nutrient solution (HP) is a mixture of hepatoprotective peptides derived from fresh porcine liver with various effects. However, the role and mechanisms of HP in nonalcoholic fatty liver disease (NAFLD) are still not well understood. We investigated the effects of HP NAFLD rats induced by high‐cholesterol diet (HCD) and its underlying mechanisms. Rats were provided with HCD for 4 weeks and then received HP or metformin after 2 weeks of HCD feeding. The study found that HP reduced cholesterol and triglyceride levels in rats with NAFLD (all *p* < .05). Histopathological examination also showed that HP improved the liver lesions induced by the HCD diet. Furthermore, the oxidative stress and inflammatory responses of NAFLD rats treated with HP were also improved. In addition, it was discovered that HP triggered the activation of AMPK and decreased the expression of SREBP‐1c and FAS while enhancing the expression of PPAR α and CPT‐1 in liver. These findings indicated that HP might have therapeutic potential for NAFLD, possibly via activating AMPK signaling pathway.

## Graphical abstract

Hepatic polypeptide nutrient solution may reduce the development of NAFLD induced by a high‐cholesterol and high‐fat diet in rats by inhibiting lipid synthesis and exerting anti‐inflammatory and antioxidative stress effects.

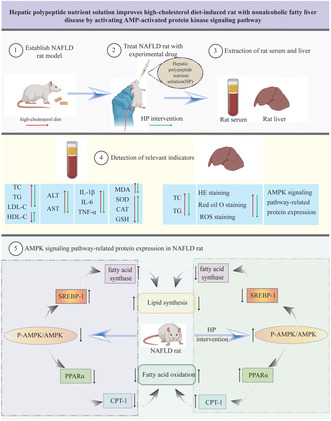

## 1 INTRODUCTION

Nonalcoholic fatty liver disease (NAFLD) is characterized by the presence of steatosis in over 5% of hepatocytes in individuals without excessive alcohol consumption or other chronic liver ailments, and it is linked to metabolic risk factors such as obesity and type 2 diabetes (Powell et al., 2021). In 2020, there was a suggestion to reclassify NAFLD as metabolic dysfunction‐associated fatty liver disease (MAFLD) (Eslam et al., 2020). The range of NAFLD covers simple steatosis to nonalcoholic steatohepatitis (NASH) with or without fibrosis (Huby & Gautier, 2022). It bears noting that NAFLD has increasingly been recognized as an underlying etiology for HCC and is estimated to affect approximately 25% of the global population. Hence, the issue of preventing and treating NAFLD has become a significant matter of public health importance (Foerster et al., 2022; Huang et al., 2021). At present, there are no established standard protocols for the pharmacological treatment of NAFLD. Thus, lifestyle intervention, including dietary improvements and weight management, remains the primary approach for its prevention and treatment (Shen & Lu, 2021; Vornoli et al., 2022). Therefore, there is growing attention toward dietary supplementation with therapeutic properties as a means of improving health, preventing diseases, and supporting overall bodily function (Sachdeva et al., 2020).

Except for the unclear pathogenesis of NAFLD, the disorder of lipid metabolism is widely regarded as an initial and critical factor (Deprince et al., 2020). Hepatic steatosis, characterized by excessive lipid deposition in hepatocytes, can be attributed to an increased influx of lipids from adipose tissues, enhanced de novo fatty acid synthesis, or impaired lipid decomposition. These processes contribute to progressing metabolic abnormalities and cellular injury (Badmus et al., 2022; Ipsen et al., 2018). In addition to lipid metabolism alterations, emerging research suggests a close interconnection between NAFLD progression, inflammation, and oxidative stress (Choudhury et al., 2011; Rector et al., 2010). AMP‐activated protein kinase (AMPK) plays a pivotal role in lipid synthesis and metabolism (Ye et al., 2022). In NAFLD, AMPK activity is diminished, while its activation can significantly improve the condition (Garcia et al., 2019). This highlights the crucial regulatory role of AMPK in NAFLD development.

López‐Pedrouso, Lorenzo, et al. have shown that porcine liver is a valuable protein source, containing approximately 18.54% protein, and is abundant in bioactive peptides that offer significant health benefits (López‐Pedrouso et al., 2021). However, the high‐fat, high‐carbohydrate, and high‐calorie content of porcine liver tissue pose certain drawbacks. To address these limitations, the research group developed a hepatic polypeptide nutrient solution (HP) using advanced biotechnology techniques. HP was extracted, isolated, and purified from porcine liver, resulting in a highly active product with zero lipid content. The oral administration of HP provides the advantages of both safety and rapid drug delivery (Du et al., 2022; Paternostro & Trauner, 2022). Studies have indicated that porcine liver hydrolysate contains a wealth of antioxidant peptides and other active substances that effectively protect the liver (López‐Pedrouso et al., 2021). Nonetheless, the precise impact of HP on improving NAFLD and its molecular mechanism of action remains incompletely understood. Therefore, the objective of this study was to establish a NAFLD rat model through the administration of a high‐cholesterol diet (HCD) and investigate the hepatoprotective effects of HP, while exploring its underlying molecular mechanism in NAFLD.

## 2 MATERIALS AND METHODS

### 2.1 Materials

Cholesterol and propylthiouracil were obtained from Shanghai Macklin Biochemical Co., Ltd. (Shanghai, China). Sodium cholate was purchased from Beijing Aoboxing Biotechnology CO., Ltd. (Beijing, China). Hebei Medical University (Shijiazhuang, China) supplied HCD and the standard rat chow. Primary antibodies, including phosphorylated AMPK (P‐AMPK) and AMPK, were supplied by Shanghai Abways Biotechnology Co., Ltd. (Shanghai, China). Peroxisome proliferator‐activated receptor alpha (PPARα), carnitine palmitoyl transferase (CPT‐1), sterol regulatory element‐binding protein (SREBP), fatty acid synthase (FAS), and GAPDH were obtained from Jiangsu Affinity Biosciences Co., Ltd. (Jiangsu, China). Secondary antibodies, including goat anti‐rabbit IgG (H‐L) HRP and goat anti‐mouse IgG (H‐L) HRP, were provided by Shanghai Abways Biotechnology Co., Ltd. (Shanghai, China).

### 2.2 Preparation of HP

HP was prepared by the research group using the following method: fresh (frozen) porcine liver that has been cut into pieces and drained after being rinsed under running water. Distilled and deionized water (ddH2O) were added to the drained liver pieces at a 1:2 ratio (m/V), followed by homogenization for 3 min, the liver solution was ultrasonicated with 1200DT ultrasonic cell crusher (Biosafer Technologies Co., Ltd., Beijing, PR China) at a power of 300 W for 15 min. These processes were repeated five times and then centrifuged to take the supernatant. The extracted supernatant was hydrolyzed with trypsin, adjusted to pH 7.5, and heated at 55°C for 2 h. Following the hydrolysis, the enzymes were inactivated by heating at 100°C for 15 min. The hydrolysate was centrifuged at 2000*g* for 15 min at 4°C, and an appropriate amount of diatomaceous earth and activated carbon was added and adsorbed for 1 h, then filtered to obtain the filtrate. After prefiltering the filtrate with a 1‐μm filter to obtain the filtrate, the prefiltrate underwent 10 kD ultrafiltration, was filtered, and the filtrate was collected to obtain HP.

The protein content in HP was determined according to the Kjeldahl method (Zhao et al., 2022). A high‐performance liquid chromatography (HPLC) method was used to determine the free amino acid content of HP, and the content of polypeptide was analyzed according to GB/T 4588.

### 2.3 Experimental procedures for animals

Healthy 8‐week‐old male Sprague–Dawley (SD) rats weighing 200 ± 10 g were obtained from Liaoning Changsheng Biotechnology Co., Ltd [Certificate No. SCXK (Liao) 2020–0001]. The rats were specific pathogen‐free (SPF) and raised within the Experimental Animal Center of the Hebei University of Chinese Medicine. They were kept in standard cages under controlled conditions with a temperature of 24 ± 2°C and provided with ad libitum access to food and water. The Animal Care and Ethical Committee of the Hebei University of Chinese Medicine approved the animal studies conducted in this research (approval number: DWLL202203133). All experimental procedures adhered to the principles and guidelines outlined within the 1996 National Institutes of Health Guide for the Care and Use of Laboratory Animals.

Five groups (*n* = 7) of 35 male SD rats were randomly divided after 1 week of acclimation: control (NC), model (MOD), low‐dose HP (HP‐L), high‐dose HP (HP‐H), and metformin (MET) groups. The rats in the NC group were exclusively fed a standard rat chow, while the rats in the other groups were fed an HCD for a duration of 4 weeks, which consisted of 94.5% standard chow, 4% cholesterol, 1% sodium cholate, and 0.5% propylthiouracil (Elseweidy et al., 2016). After 2 weeks of HCD feeding, the rats in each group received their respective treatments. HP was administered by gavage at doses of 40 mg/kg/day in the HP‐L group and 120 mg/kg/day in the HP‐H group. Metformin (Sino‐American Shanghai Squibb Pharmaceuticals Ltd. Shanghai, China) was given to the MET group at 150 mg/kg/day (Feng et al., 2019). Normal saline was administered in an isovolumic manner to both the NC and MOD groups. The HP‐L dose was chosen to reflect the equivalent clinical dose in humans based on an average body weight of 60 kg/day in adults (Nair et al., 2018). Weekly body weight measurements were regularly monitored throughout the experimental period.

After a 12‐h fasting period, the rats' final weight was recorded on the 29th day, then they were anesthetized with 40 mg/kg pentobarbital intraperitoneally. Through centrifugation, serum was separated from blood samples drawn from the femoral artery. Additionally, liver tissue was collected from each rat to assess relevant indicators. The liver was visually inspected, subsequently removed, and weighed. For the experiment, a solution of 4% paraformaldehyde was utilized to conserve a portion of the liver, whereas the rest of the liver tissue was frozen in liquid nitrogen and stored at a temperature of −80°C for subsequent examination.

### 2.4 Detection of biochemical indicators in serum

Commercially available kits from Jian Cheng Biological Engineering Institute (Nanjing, China) were utilized to measure various parameters in serum, including triglycerides (TG), total cholesterol (TC), high‐density lipoprotein cholesterol (HDL‐C), low‐density lipoprotein cholesterol (LDL‐C), aspartate aminotransferase (AST), aminotransferase (ALT), malondialdehyde (MDA), superoxide dismutase (SOD), glutathione (GSH), and catalase (CAT).

### 2.5 Detection of inflammatory cytokines in serum

Serum tumor necrosis factor (TNF)‐α, interleukin (IL)‐1β, and IL‐6 were measured using enzyme‐linked immunosorbent assay kits, following the manufacturer's guidelines from MultiSciences Biotech Co., Ltd. (Hangzhou, China).

### 2.6 Detection of TC and TG in liver

Liver tissue was homogenized to measure lipid levels, following the instructions provided in the kit. Each liver tissue group was weighed and homogenized in anhydrous ethanol at a 1:9 weight‐to‐volume ratio. The supernatant collected after 10 min of centrifugation at 2500 rpm was used for analysis. Subsequently, the levels of TC and TG in the liver were determined using the same kit utilized for serum analysis.

### 2.7 The liver index and histopathological examination of the liver

The liver index has been measured on the day of sacrifice as absolute liver weight (g) divided by body weight (g) and multiplied by 100% (Zhou et al., 2022).

Liver pathology evaluation was performed using hematoxylin–eosin (HE) staining and oil red O staining. A consistent fixation was achieved by immersing the same portion of each rat's liver tissue in 4% paraformaldehyde for over 48 h, followed by embedding in paraffin. The tissue was then sectioned into 4‐μm‐thick slices and stained with HE for observation. Similarly, 5‐μm sections of frozen liver tissues were prepared and subjected to oil red O staining following the recommended procedure provided by the kit. The structures and lipid droplets within the liver were visualized by light microscopy under 200× magnification (Leica DM4000B, Solms, Germany) after both HE staining and oil red O staining. The individual's NAFLD activity score (NAS) was determined, which involved a semiquantitative evaluation of four aspects: steatosis (0–3), lobular inflammation (0–2), hepatocellular ballooning (0–2), and fibrosis (0–4) using a semiquantitative method. A diagnosis of NAFLD was made if the total score exceeded 3 (Kleiner et al., 2005). The lipid droplets stained with oil red O were analyzed using ImageJ software (Bethesda, USA). Each sample was photographed with five randomly selected images. The data were collected from at least three independent experiments. Quantitative analysis of the lipid droplets stained with oil red O was performed using ImageJ software (Bethesda, USA). Each sample was subjected to photography, capturing five randomly selected images. The data were obtained from a minimum of three independent experiments.

### 2.8 Dihydroethidium (DHE) probe detection of reactive oxygen species (ROS) in liver

We first thawed the frozen sections to room temperature using a liquid barrier pen to mark the target tissue. The tissue was subsequently subjected to incubation with a reagent capable of spontaneous fluorescence quenching for a duration of 5 min, followed by rinsing with running tap water for a period of 10 min. Afterward, a ROS staining solution was administered to the specified site, followed by incubation at a temperature of 37°C without exposure to light for 30 min. The slides were then washed with PBS and subjected to incubation with a DAPI solution at room temperature in the absence of light for a duration of 10 min. The aforementioned washing procedure was then repeated for liver tissue sections, after which they were dried and sealed using an antifade mounting medium (Zou et al., 2021). Photographic images were captured using fluorescent microscopy (Nikon Eclipse C1, Nikon, Japan).

### 2.9 Western blot

After homogenizing liver tissues in RIPA lysis buffer (Servicebio Technology Co., Ltd, Wuhan, China) under ice bath, centrifugation for 30 min at 12,000 rpm under 4°C was carried out to collect the supernatant. The bicinchoninic acid (BCA) protein assay kit (Beijing Cowin Biotech Co., Ltd., Beijing, China) was employed to determine the total protein concentration. Protein samples, of equal quantities, were subjected to 10% sodium dodecyl sulfate–polyacrylamide gel electrophoresis and subsequently transferred to polyvinylidene difluoride membranes. After blocking with 5% skimmed milk for 2 h at room temperature, the membranes were incubated overnight at 4°C with different primary antibodies: P‐AMPK, AMPK, SREBP, CPT‐1, PPARα, FAS at 1:1000 dilution, and GAPDH at 1:5000 dilution. The membranes were washed, shaken for 1 h in the suitable secondary antibody, washed again, and then incubated in enhanced chemiluminescence solution for subsequent detection (Yeasen Biotechnology Co., Ltd., Shanghai, China). The protein bands were scanned and analyzed with Vision Capt software (Kunming, China).

### 2.10 Statistical analyses

All data were presented as mean ± standard deviation using GraphPad Prism 9.0.0 (San Diego, USA) by one‐way ANOVA followed by Tukey's test. Statistical significance was defined as *p* < .05.

## 3 RESULTS

### 3.1 The protein, free amino acid, and polypeptide content of HP

The free amino acid analysis revealed that leucine was the predominant amino acid in HP, followed by lysine, phenylalanine, arginine, and valine. Additionally, the protein content (in terms of total nitrogen) in HP was 16.62 mg/mL, with a polypeptide content of 69% (Table 1).

**TABLE 1:** The protein, free amino acid, and polypeptide content of the hepatic polypeptide nutrient solution.

	Hepatic polypeptide nutrient solution
Protein (as total nitrogen) (mg/mL)	16.62
Free amino acids (mg/mL)	
Aspartic acid	0.47
Glutamic acid	2.29
Phenylalanine	2.85
Threonine	1.28
Tyrosine	2.13
Serine	1.78
Methionine	1.38
Tryptophan	0.50
Valine	2.65
Glycine	0.96
Leucine	5.91
Alanine	2.32
Isoleucine	1.63
Proline	0.76
Histidine	1.25
Lysine	4.39
Arginine	2.83
Polypeptide content (%)	69%

### 3.2 Effect of HP on the general status of rats

**FIGURE 1:**
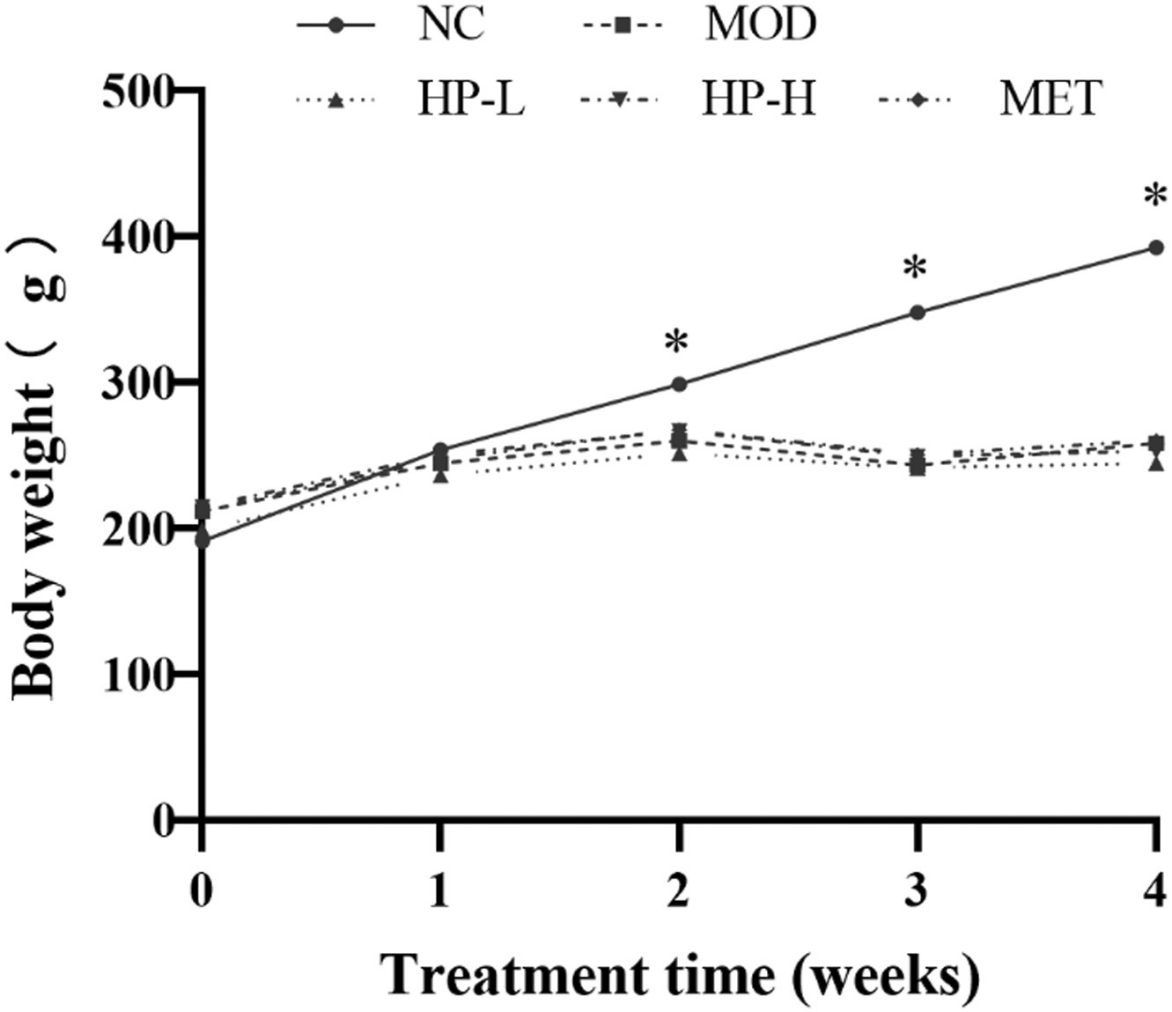
Effect of HP on general status of rats. The samples were obtained from the normal control group (NC), model group (MOD), the low‐dose HP‐treated group (HP‐L), the high‐dose HP‐treated group (HP‐H), and the metformin group (MET). Data were presented as the mean ± SD (*n* = 7). *p < .05 versus NC, and ^#^ *p* < .05 versus MOD.

No significant fluctuations were observed in the vital signs of either group of rats during the experimental period. Dull fur coloration was observed in the rats of the MOD group, whereas the other groups of rats presented normal fur coloration and mobility. There was a notable distinction in body weight between the NC and MOD groups (*p* < .05). Nevertheless, the treatment and MOD groups did not differ significantly in body weight (Figure 1).

### 3.3 Effects of HP on lipids level and liver function in serum

**FIGURE 2:**
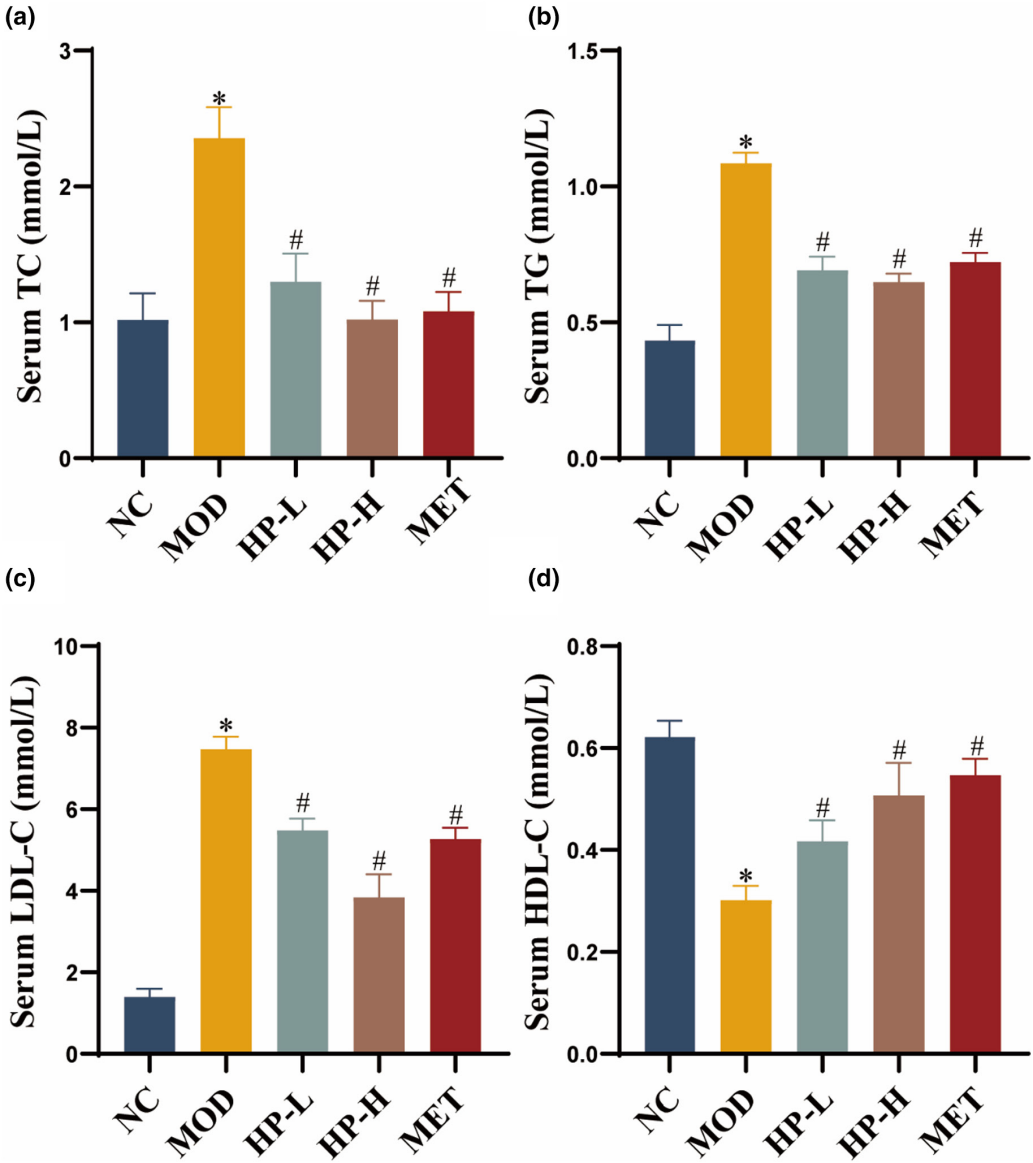
Effects of HP on lipids level in serum. Serum levels of (a) TC, (b) TG, (c) LDL‐C, and (d) HDL‐C. Serum samples were obtained from the normal control group (NC), model group (MOD), the low‐dose HP‐treated group (HP‐L), the high‐dose HP‐treated group (HP‐H), and the metformin group (MET). Data were presented as the mean ± SD (*n* = 7). *p < .05 versus NC, and ^#^ *p* < .05 versus MOD.

Serum TG, TC, and LDL‐C were elevated, whereas HDL‐C was decreased in the MOD group as compared with the NC group (all *p* < .05). Surprisingly, treatment with HP resulted in a significant reduction in TC, TG, and LDL‐C, together with a substantial increase in HDL‐C against the MOD group (all *p* < .05), and the higher doses of HP exhibited greater effectiveness (Figure 2).

**FIGURE 3:**
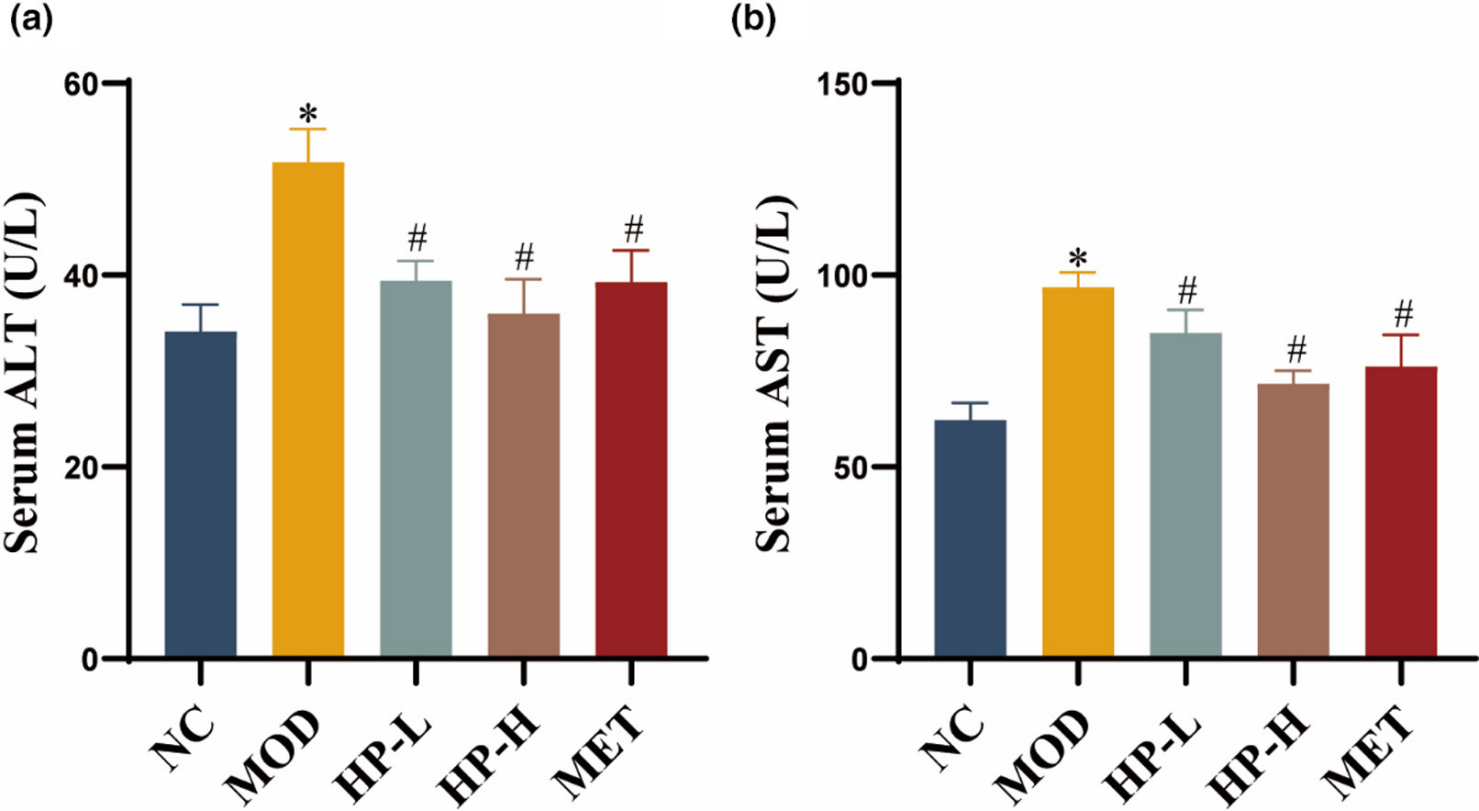
Effects of HP on liver function. (a) Serum levels of ALT activity. (b) Serum levels of AST activity. Serum samples were obtained from the normal control group (NC), model group (MOD), the low‐dose HP‐treated group (HP‐L), the high‐dose HP‐treated group (HP‐H), and the metformin group (MET). Data were presented as the mean ± SD (*n* = 7). *p < .05 versus NC, and ^#^ *p* < .05 versus MOD.

Serum levels of ALT and AST are crucial indicators reflecting liver functionality. In Figure 3, the MOD group demonstrated a clear increase in ALT and AST activities against the NC group (both *p* < .05), while the HP‐L, HP‐H, and MET groups exhibited lower AST and ALT activities compared to the MOD group (all *p* < .05).

### 3.4 Effects of HP on lipids level in liver

**FIGURE 4:**
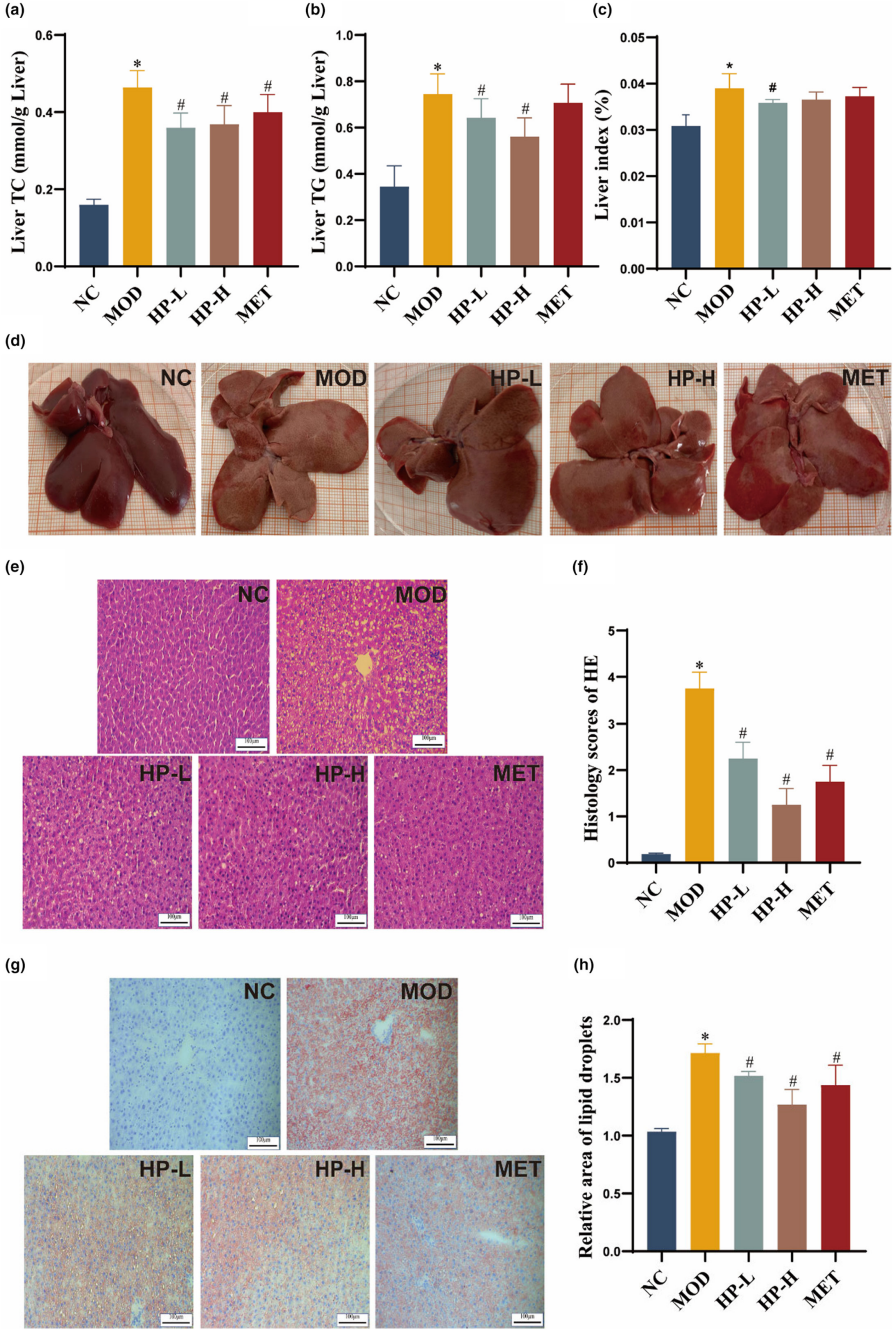
Effects of HP on lipids level and histological changes in the liver. (a) Hepatic levels of TC. (b) Hepatic levels of TG. (c) liver index. (d) Representative photos of liver. (e) Representative liver photos of HE staining (200×, scale bar =100 μm). (f) Histological scores of HE. (g) Representative liver photos of oil red O staining (200×, scale bar = 100 μm). (h) Relative area of lipid droplets. The liver samples were obtained from the normal control group (NC), model group (MOD), the low‐dose HP‐treated group (HP‐L), the high‐dose HP‐treated group (HP‐H), and the metformin group (MET). Data were presented as the mean ± SD (*n* = 7). *p < .05 versus NC, and ^#^ *p* < .05 versus MOD.

When compared to the NC group, TC and TG levels in liver tissues of the MOD group were increased, but decreased after drug administration (Figure 4a,b). In Figure 4c, the liver index in the MOD group was higher than that in the NC group (*p* < .05), while the HP‐L group demonstrated a significantly lower liver index compared to the MOD group (*p* < .05). No significant differences were observed in liver index among the MOD, HP‐H, and MET groups.

### 3.5 Effects of HP on histological changes in liver

Macroscopic observations revealed that the liver in the NC group appeared reddish‐brown with a smooth surface, soft and elastic texture, and a dense structure. However, rats in the MOD group had yellowish livers with scattered fatty spots visible on the surface, a brittle texture, increased liver volume, and a loose‐cut surface. In comparison, rats treated with HP or MET exhibited improved liver color, morphology, texture, and palpation compared to the MOD group (Figure 4d).

Histopathological examination of hepatic sections from rats in different groups was performed using HE staining and oil red O staining. The results of HE staining showed that the livers in the NC group displayed normal architecture, characterized by hepatocytes with healthy nuclei and intact parenchymal structure. However, hepatic sections from the MOD group exhibited visible fatty changes, unclear boundaries of hepatic lobules, the disorganized structure of hepatic cords, and a multitude of diffuse and mixed vacuoles in the liver tissue. The damaged area was dramatically reduced, the vacuoles were reduced, and the cellular and hepatic lobular structures were restored to varying degrees in each intervention group compared with the MOD group. The results of oil red O staining showed that liver tissue sections from the NC group exhibited normal histopathology. In the liver tissue sections from the MOD group, diffused and granular lipid droplets were observed. In contrast, the HP and MET groups displayed significantly reduced hepatocellular lipid droplet accumulation compared to the MOD group. The histopathological scores of HE and the relative area of lipid droplets with oil red O staining exhibited similar trends (Figure 4e–h).

### 3.6 Effects of HP on inflammatory cytokines in serum

**FIGURE 5:**
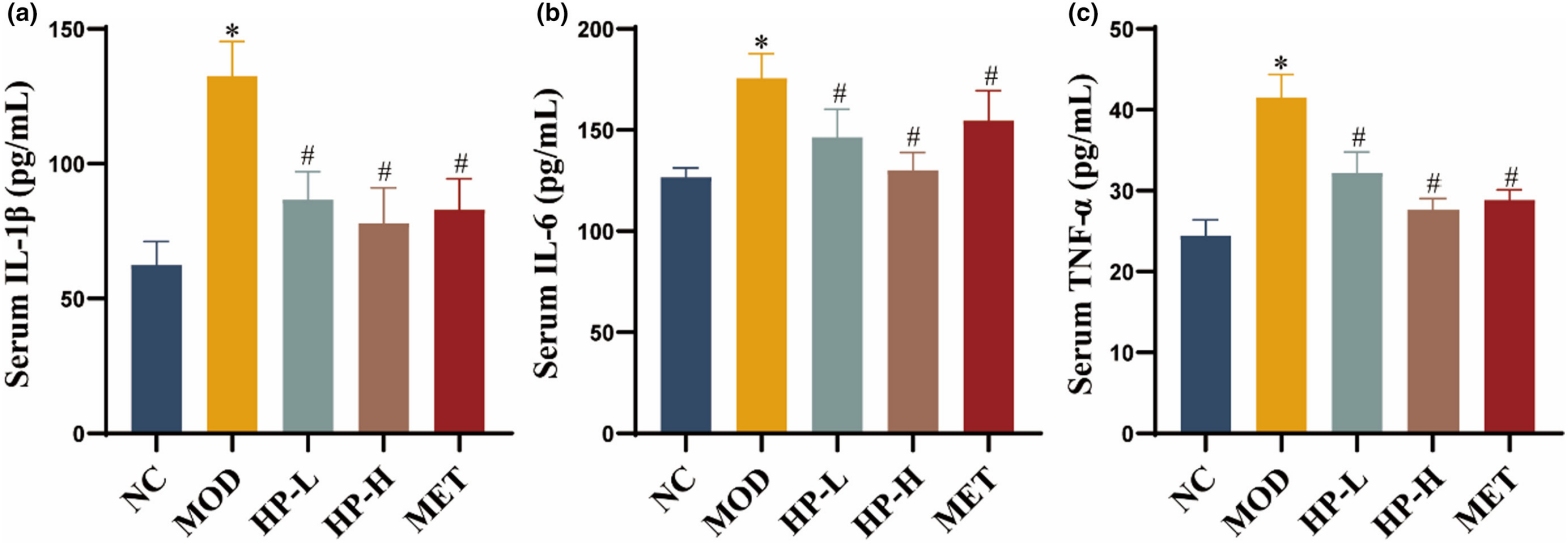
Effects of HP on inflammatory cytokines in serum. (a) IL‐1β. (b) IL‐6. (c) TNF‐α. Serum samples were obtained from the normal control group (NC), model group (MOD), the low‐dose HP‐treated group (HP‐L), the high‐dose HP‐treated group (HP‐H), and the metformin group (MET). Data were presented as the mean ± SD (*n* = 7). *p < .05 versus NC, and ^#^ *p* < .05 versus MOD.

The MOD group showed significantly elevated levels of IL‐1β, IL‐6, and TNF‐α compared to the NC group (all *p* < .05). Conversely, the HP‐L, HP‐H, and MET groups exhibited significantly reduced levels of IL‐1β, IL‐6, and TNF‐α compared to the MOD group (all *p* < .05) (Figure 5a–c).

### 3.7 Effects of HP on oxidative stress indicators in serum and liver

**FIGURE 6:**
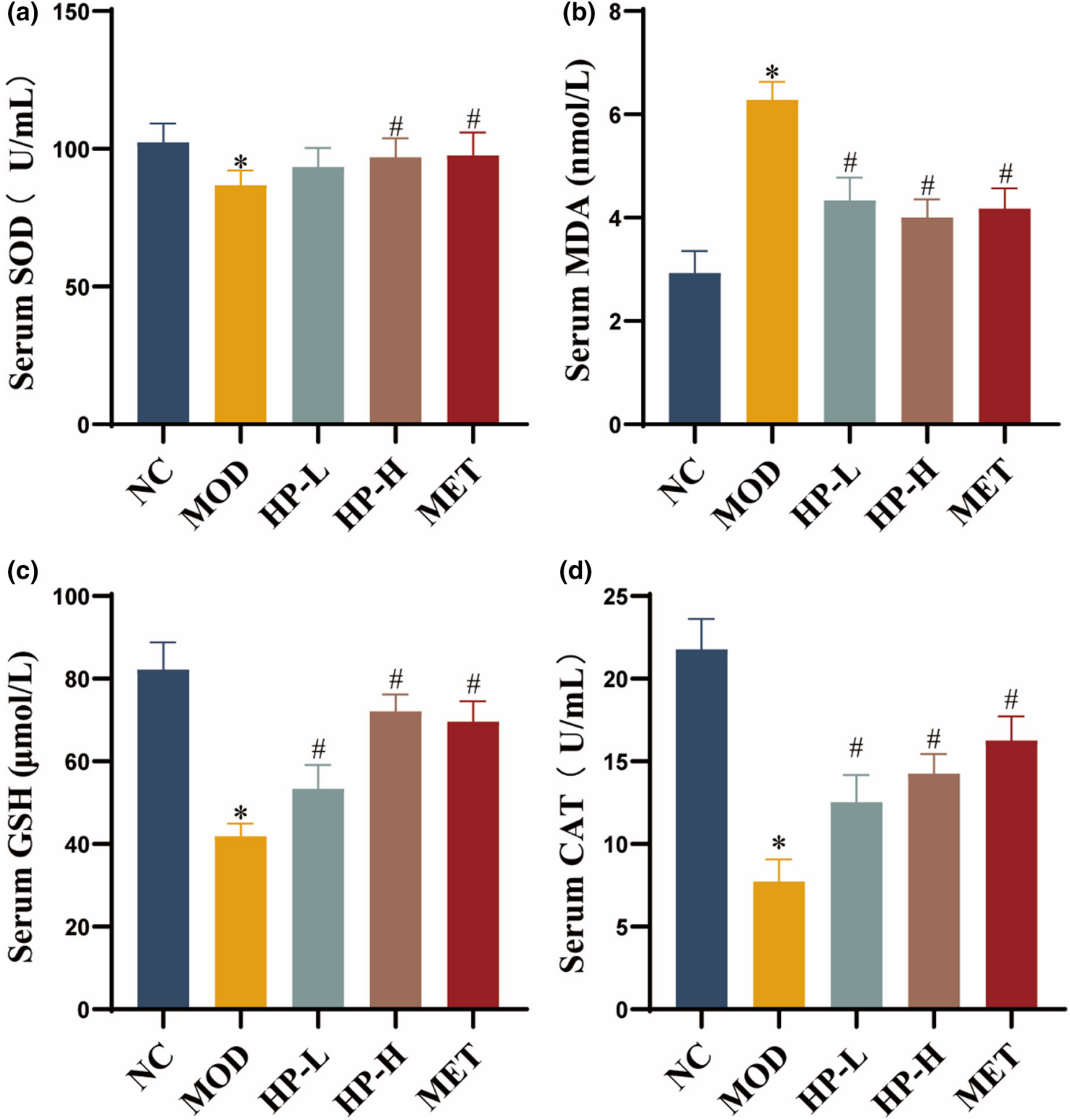
Effects of HP on oxidative stress indicators in serum. (a) Serum SOD activity. (b) Serum MDA content. (c) Serum CAT content. (d) Serum GSH content. Serum samples were obtained from the normal control group (NC), model group (MOD), the low‐dose HP‐treated group (HP‐L), the high‐dose HP‐treated group (HP‐H), and the metformin group (MET). Data were presented as the mean ± SD (*n* = 7). *p < .05 versus NC, and ^#^ *p* < .05 versus MOD.

A significant reduction of CAT, SOD, and GSH levels in serum was observed in the MOD group when compared with the NC group (all *p* < .05). There was no significant difference in serum SOD activity between the HP‐L and MOD groups. Additionally, the serum levels of CAT, SOD, and GSH from the HP‐H and MET groups were higher than those in the MOD group (all *p* < .05). Serum MDA content from MOD group showed a remarkable increase compared with NC group (*p* < .05), while in treated groups, these levels were noticeably reduced versus the MOD group (both *p* < .05) (Figure 6a–d).

**FIGURE 7:**
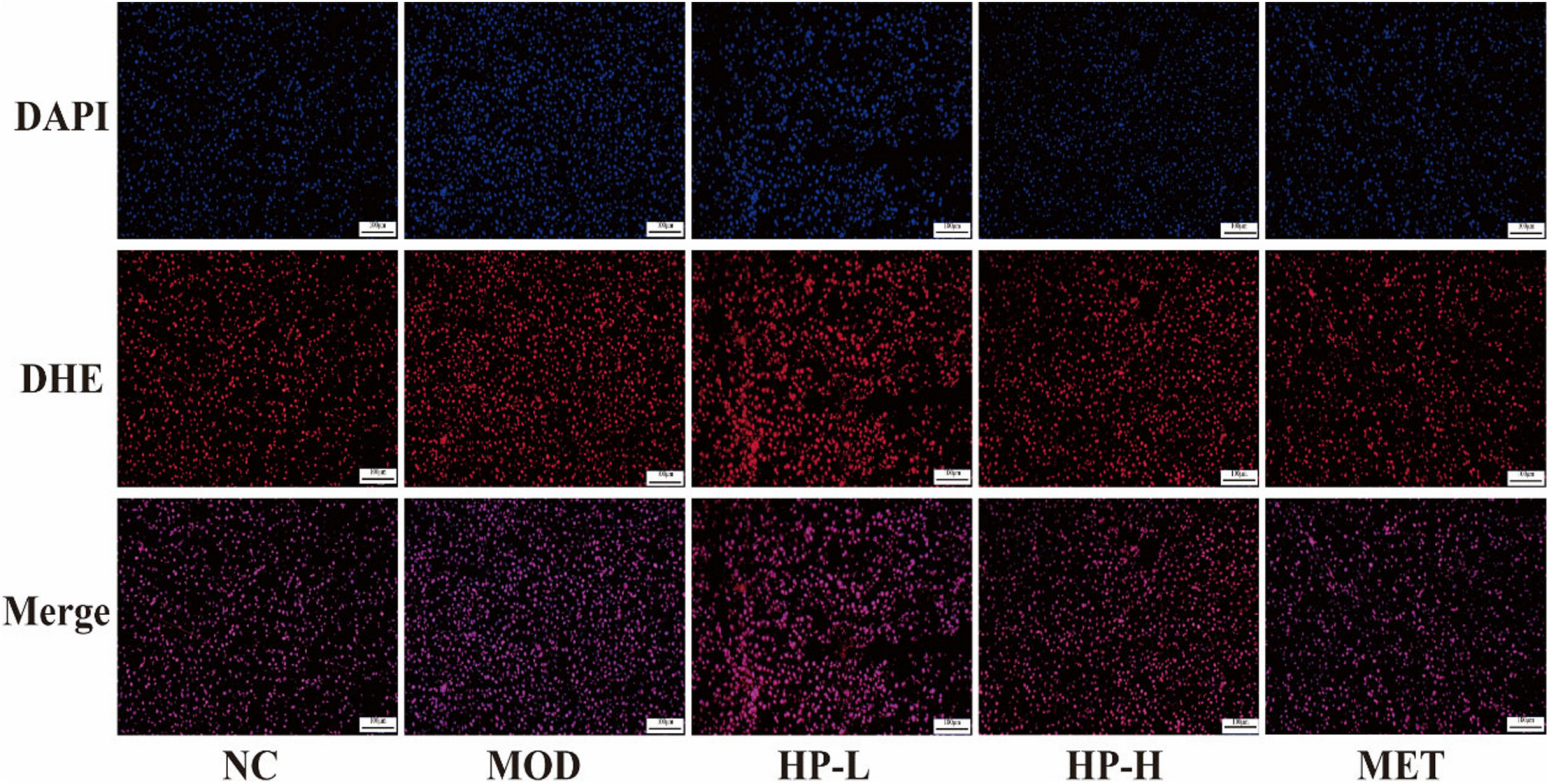
Effects of HP on oxidative stress indicators in liver. Representative reactive oxygen species (ROS) histologic slices in five groups, (200×, scale bar = 100 μm). Liver samples were obtained from the normal control group (NC), model group (MOD), the low‐dose HP‐treated group (HP‐L), the high‐dose HP‐treated group (HP‐H), and the metformin group (MET). Data were presented as the mean ± SD (*n* = 7). *p < .05 versus NC, and ^#^ *p* < .05 versus MOD.

Assessment of ROS expression in liver tissue using the DHE probe revealed that the MOD group exhibited markedly greater fluorescence intensity than the NC group. Importantly, under HP treatment, a considerable reduction in fluorescence intensity was observed versus the MOD group, with the effect being more pronounced in the HP‐H group (Figure 7).

### 3.8 Effect of HP on AMPK signaling pathway in liver

**FIGURE 8:**
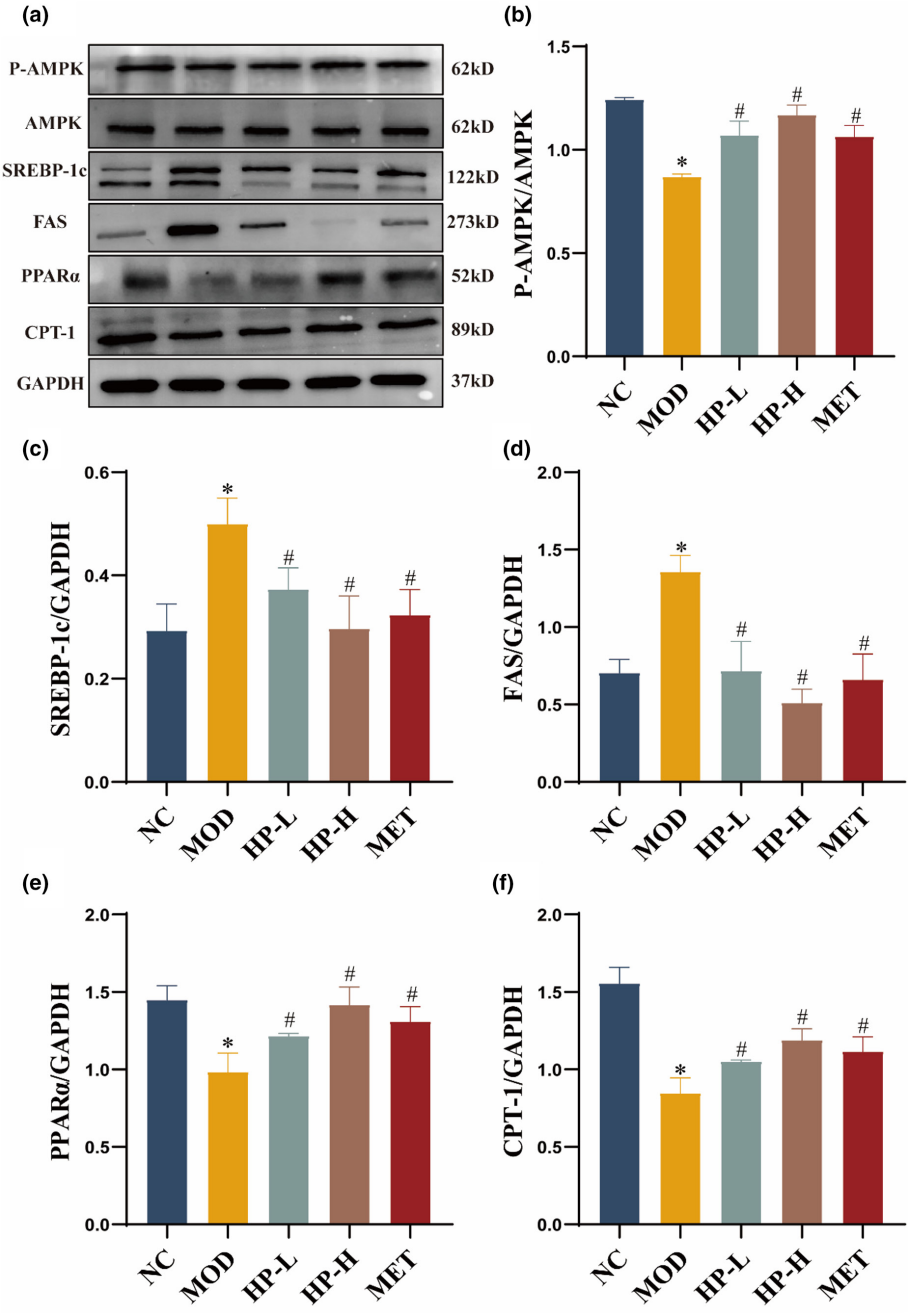
Effects of HP on AMPK, FAS, SREBP‐1c, CPT1, and PPARα expression in the liver. (a) Representative p‐AMPK, FAS, SREBP‐1c, CPT1, and PPARα expression brands. (b–f) Gray value of western blot bands. The liver samples were obtained from the normal control group (NC), model group (MOD), the low‐dose HP‐treated group (HP‐L), the high‐dose HP‐treated group (HP‐H), and the metformin group (MET). Data were presented as the mean ± SD (*n* = 7). *p < .05 versus NC, and ^#^ *p* < .05 versus MOD.

According to the findings presented in Figure 8, the treatment groups demonstrated a notable augmentation in the protein expression of CPT‐1, PPARα, and phosphorylation of AMPK versus the MOD group. Additionally, the expression levels of SREBP‐1c and FAS were notably upregulated in the MOD group when compared to the NC group, whereas, in treatment groups, SREBP‐1c and FAS expression were markedly downregulated in comparison with the MOD group (all *p* < .05).

## 4 DISCUSSION

It has been observed that HP can protect the liver; however, the specific efficacy and inherent mechanisms of HP treatment in NAFLD rats have not been clearly defined. This study aimed to examine the efficacy of HP in improving NAFLD rats, focusing on lipid synthesis, inflammatory and oxidative stress, and the AMPK signaling pathway. It is suggested by our data that HP might reduce the development of HCD diet‐induced NAFLD rats by inhibiting lipid synthesis as well as exerting effects on anti‐inflammatory and antioxidative stress. Furthermore, the mechanism might be associated with the activation of AMPK signaling pathway at least in part.

NAFLD encompasses various degrees of liver damage, including simple steatosis, fibrosis, and cirrhosis as described by Nassir (2022). Hypercholesterolemia has been identified as a prominent risk factor for the onset of NAFLD, as highlighted in the research by Pirillo et al. (2021). Consequently, regulating plasma cholesterol levels and preventing hypercholesterolemia are commonly employed strategies to control the development of NAFLD. In our research, we established a NAFLD model in rats by subjecting them to a 4‐week HCD diet. Previous studies have demonstrated that rats fed with HCD experience severe liver damage and exhibit significantly elevated serum lipid levels (Gao et al., 2019). Consistent with these findings, rats fed HCD for 4 weeks showed raised serum TG, TC, and LDL‐C, accompanied by decreased HDL‐C in the present study. Histological analysis revealed altered liver structure with significant lipid deposition in HCD diet rats, which aligns with the earlier discoveries of Jahn et al. (2019). Accordingly, the NAFLD rat model established in this experiment was successful.

MET was known to prevent the development of diet‐induced NAFLD and activate AMPK, so we selected it as the positive control drug in this experiment (Lv & Guo, 2020). Following HP intervention, the rats in the HP group showed a notable decrease in serum lipid levels, such as TG, TC, and LDL‐C, compared to those in the MOD group. Furthermore, the serum ALT and AST were dramatically decreased by HP intervention. These results indicated that HP intervention could improve serum lipid levels and liver function in NAFLD rats. Additionally, HE staining and oil red O staining were used to assess the pathological morphology of the liver and determine the liver lipid content. These results demonstrated decreased lipid deposition and improved liver structure following HP treatment.

Inflammation and oxidative stress are recognized as significant factors in the progression of NAFLD. In normal liver tissue, there exists a dynamic balance between oxidative and antioxidant systems. The liver efficiently neutralizes physiological ROS through intracellular antioxidant mechanisms. However, in NAFLD, ROS levels are excessively produced due to impaired mitochondrial ROS production and ROS scavenging mechanisms under pathological conditions. This results in the production of lipid peroxides such as MDA, a toxic by‐product of oxidative stress (Lee et al., 2019). Consequently, the excessive production of ROS and lipid peroxides can result in hepatocyte damage. Moreover, ROS accumulation stimulates the production of inflammatory cytokines, including IL‐1β, IL‐6, and TNF‐α. The secretion of inflammatory cytokines activates liver resident macrophages (Kupffer cells) and hepatic stellate cells, leading to the recruitment of inflammatory cells and liver fibrosis, thereby promoting the progression from simple fatty liver to nonalcoholic steatohepatitis (NASH) (Li et al., 2021; Schwabe et al., 2020). Therefore, the inhibition of liver oxidative stress and ROS production is crucial in the prevention and treatment of NAFLD. In the present research, various indicators of oxidative stress and inflammation were examined. Our results indicate that HP treatment decreased hepatic ROS levels and serum MDA content while increasing serum SOD, CAT activity, and GSH contents as markers of oxidative stress. Furthermore, HP treatment reduced the inflammatory cytokines in serum of NAFLD rats. Consequently, HP treatment in rats with NAFLD might effectively reduce oxidative stress and inflammatory responses.

As widely recognized, AMPK is a well‐established therapeutic target for NAFLD (Chyau et al., 2020; Zhang et al., 2009). Phosphorylated AMPK is considered one of the most important intracellular energy sensors that can reverse NAFLD by regulating fatty acid synthesis, enhancing mitochondrial utilization and oxidation of fatty acids, and improving liver inflammation and fibrosis (Fang et al., 2022). Research indicates that increased hepatic lipogenesis in NAFLD patients is associated with activating SREBP‐1c, a transcription factor that plays a major role in regulating lipogenic proteins such as FAS. Overexpression of SREBP‐1c has been shown to induce hepatocyte steatosis, and AMPK is responsible for controlling SREBP‐1c activity in the liver (Fang et al., 2022; Kawamura et al., 2022; Liu et al., 2023). PPARα, a ligand activator, controls various aspects of lipid catabolism and serves as a major regulator of lipid oxidation in the liver, thus reducing hepatic lipid levels (Jia et al., 2013; Rakhshandehroo et al., 2010).

Additionally, CPT‐1, a key regulator of fatty acid oxidation, is closely linked to the process of fatty acid oxidation (Liu et al., 2011). AMPK activation also upregulates PPARα and its downstream targets, including CPT‐1, thereby promoting fatty acid oxidation (Diniz et al., 2021; Ma et al., 2022). In our study, we observed elevated phosphorylation levels of AMPK and changes in the expression of its downstream molecules, including SREBP‐1c, PPARα, CPT‐1, and FAS, following HP treatment in HCD‐fed NAFLD rats. These results suggested that HP might exert a beneficial effect on liver steatosis through targeted activation of the AMPK signaling pathway.

## 5 CONCLUSION

Overall, our results suggested that the consumption of HP could provide numerous health benefits, including antioxidant, anti‐inflammatory, and lipid‐lowering effects, which make it a potentially valuable dietary intervention for promoting overall well‐being. These effects might be achieved through the upregulation of p‐AMPK, CPT1, and PPARα, as well as the downregulation of SREBP‐1c and FAS pathways. However, while our study highlights the beneficial effects of HP consumption, it does not specifically identify the components of HP responsible for these effects. Further research is necessary to isolate and study the individual components of HP in order to elucidate their specific contributions to the observed outcomes. Additionally, conducting cellular studies would allow for a more comprehensive understanding of the underlying mechanisms and provide additional evidence to support our findings.

## AUTHOR CONTRIBUTIONS

**Yingying Xiao:** Data curation (equal); investigation (equal); writing – original draft (equal). **Jianan Wang:** Data curation (equal); resources (equal). **Ying Zhang:** Data curation (equal); resources (equal); software (equal). **Ting Zhang:** Formal analysis (equal); investigation (equal). **Xingzhong Qi:** Formal analysis (equal); investigation (equal). **Lei Hou:** Formal analysis (equal). **Zhihong Ma:** Conceptualization,Supervision, Funding acquisition, Experimental guidance, Project administration. **Feng Xu:** Conceptualization (equal); funding acquisition (equal); methodology (equal).

## CONFLICT OF INTEREST STATEMENT

Zhihong Ma, Yingying Xiao, Jianan Wang, Ying Zhang, Ting Zhang, Xingzhong Qi, Lei Hou: no competing interests declared. Xu Feng is a full‐time executive employee of Hebei Zhitong Biological Pharmaceutical Co., Ltd. This affiliation has no effect on his contribution. Besides this, other conflicts of interest do not exist.

## Data Availability

The data utilized in this paper can be made accessible upon request by the reviewer or editor.
